# Covalent conjugation of proteins onto fluorescent single-walled carbon nanotubes for biological and medical applications

**DOI:** 10.1039/d2ma00714b

**Published:** 2022-11-29

**Authors:** Hanxuan Wang, Ardemis A. Boghossian

**Affiliations:** a Ecole Polytechnique Fédérale de Lausanne (EPFL), Institute of Chemical Sciences and Engineering CH-1015 Lausanne Switzerland ardemis.boghossian@epfl.ch

## Abstract

Single-walled carbon nanotubes (SWCNTs) have optical properties that are conducive for biological applications such as sensing, delivery, and imaging. These applications necessitate the immobilization of macromolecules that can serve as therapeutic drugs, molecular templates, or modulators of surface interactions. Although previous studies have focused on non-covalent immobilization strategies, recent advances have introduced covalent functional handles that can preserve or even enhance the SWCNT optical properties. This review presents an overview of covalent sidewall modifications of SWCNTs, with a focus on the latest generation of “sp^3^ defect” modifications. We summarize and compare the reaction conditions and the reported products of these sp^3^ chemistries. We further review the underlying photophysics governing SWCNT fluorescence and apply these principles to the fluorescence emitted from these covalently modified SWCNTs. Finally, we provide an outlook on additional chemistries that could be applied to covalently conjugate proteins to these chemically modified, fluorescent SWCNTs. We review the advantages of these approaches, emerging opportunities for further improvement, as well as their implications for enabling new technologies.

## Abstract 2

Recent advances enable new chemical strategies for covalent protein linking to single-walled carbon nanotubes.

## Graphical abstract

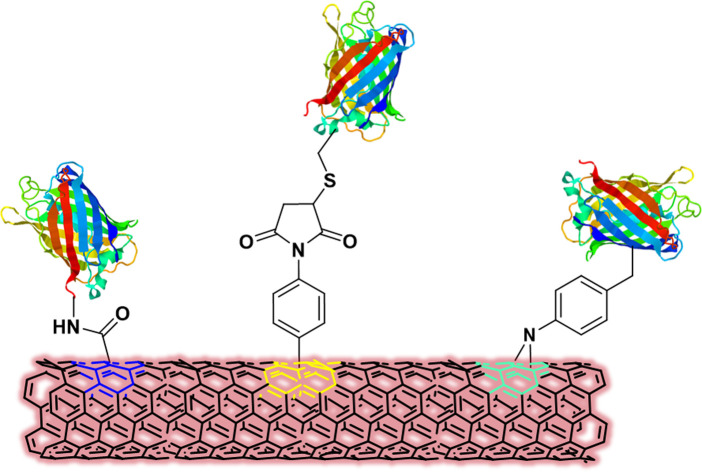

## 1. Introduction

Carbon nanotubes are molecular cylinders that consist of carbon. While they are available as multi-walled and single-walled structures, the single-walled carbon nanotubes (SWCNTs) have sharply defined properties that vary with diameter and chiral angle.^1–4^ SWCNT diameters range from 0.5–2.5 nm and may show metallic, semi-metallic, or semiconducting behavior. This tunability provides a foundation for optoelectronic applications, especially in sensing. For example, SWCNT-based electrodes have been used to detect various bio-analytes through a change in electric potential.^5–8^ These constructs rely on the immobilization of binding proteins or enzymes that trigger the change through analyte binding or catalytic activity. In addition to electronic sensors, SWCNTs can also be used as optical sensors. Semiconducting SWCNTs have band gaps that depend on the nanotube diameter and chiral angle. This variation in bandgap results in a distribution of chirality-dependent excitation and fluorescence emission peaks. The sensitivity of the fluorescence emissions to the nanotube's environment allows the SWCNT to behave as an optical modulator. As with electronic sensors, analyte binding often relies on protein conjugation to the nanotube surface.^9,10^

**Fig. 1:**
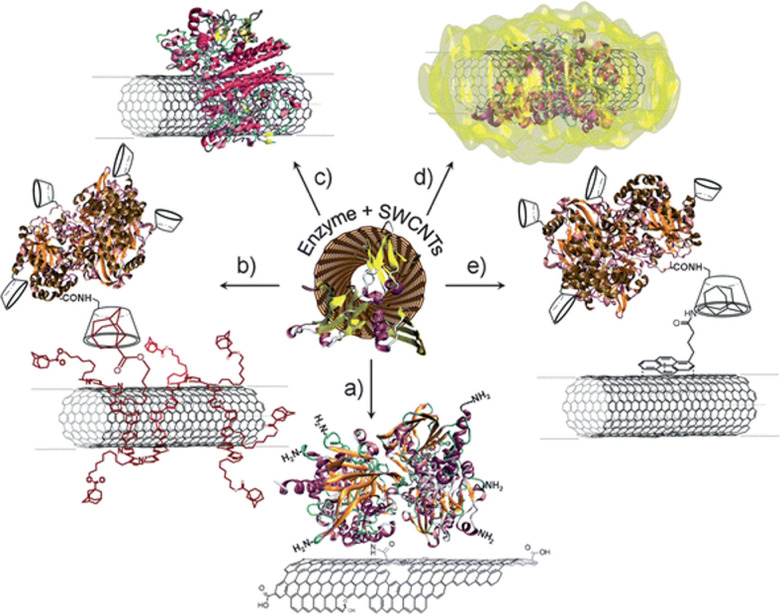
Immobilization strategies of enzymes on SWCNTs: (a) covalent binding *via* amide coupling with the carboxylic acid groups of oxidized nanotubes; (b) electrochemical coating of nanotubes with affinity partners and subsequent immobilization of affinity counter-part modified enzymes; (c) adsorption of enzymes on SWCNTs *via* hydrophobic or electrostatic interactions; (d) entrapment of enzymes in a polymer matrix formed around SWCNTs; and (e) immobilization *via* affinity interactions onto functionalized nanotubes. Reprinted from ref. 11, with permission from The Royal Society of Chemistry.

Protein conjugation is therefore a critical component that is shared across different sensing configurations. This conjugation can be achieved using several approaches (Fig. 1).^11^ The most common approach for pristine nanotubes is immobilization through non-covalent interactions such as π–π, hydrophobic, and van der Waals interactions (Fig. 1c).^12–14^ Although the preparation is relatively facile, the resulting conjugates often have uneven protein loading with heterogeneous configurations. Separation and purification of these conjugates remain an ongoing challenge.^15,16^ Non-covalent electrochemical coatings (Fig. 1b) and linkers (Fig. 1e) can be used to further control protein orientation. However, as with non-specific adsorption, these approaches rely on non-covalent interactions that are less stable compared to covalent bonds. The immobilized proteins are also more prone to denaturation and degradation.^17,18^ Enzyme entrapment can be used to stabilize the protein onto the nanotube surface (Fig. 1d), though often at the cost of enzyme accessibility to the analyte.

Covalent conjugation (Fig. 1a) presents an alternative approach to immobilizing proteins in a controlled, stabilized, and accessible manner.^19^ Until recently, these strategies have been largely limited to electronic sensors; the covalent perturbation of the carbon lattice often results in fluorescence quenching that limits the application of the SWCNTs for optical sensing. However, recent chemical advances now allow researchers to covalently functionalize nanotubes in a manner that sustains their fluorescence. In this review, we summarize these covalent approaches for conjugating proteins to fluorescent SWCNTs. We briefly revisit methods based on acid oxidation for introducing covalent functional handles that diminish nanotube fluorescence. We then discuss covalent approaches that preserve or even enhance SWCNT fluorescence, summarizing their applications to protein and peptide immobilization. Finally, the review concludes with the purification and application of these covalent bioconjugates.

## 2. Chemical modification of the SWCNT surface

### 2.1 Acid oxidation of SWCNTs

Covalent bioconjugation requires the introduction of functional chemical handles on the nanotube surface.^20,21^ The most common approach relies on carboxylic acid functional handles. In this approach, a strongly oxidizing acid (HNO_3_, H_2_SO_4_, mixture of HNO_3_ and H_2_SO_4_ or mixture of KMnO_4_ and H_2_SO_4_) is added to the SWCNTs to break the carbon sp^2^ bonds on the SWCNT ends and sidewalls.^22–24^ This oxidative reaction introduces several functional groups that include not only carboxylic groups but also hydroxyl and acyl groups. The carboxylic groups serve as the primary reactive sites for the subsequent immobilization steps.^25^ The direct amino conjugation to the protein is commonly achieved through a condensation reaction with the carboxylic group that forms an amide bond. Since the yield of this step is usually limited by the steric hindrance between the amino residues,^26^ the loading of the carboxylic groups, though poorly controlled, is often sufficient for protein immobilization.

Though the acidification functionalization benefits from relatively high oxidative yields and reaction simplicity, several disadvantages limit its use for optical sensing. First, the harsh reaction conditions contribute to SWCNT fragmentation and shortening.^27,28^ Since the nanotube ends act as exciton quenching sites, this shortening decreases the nanotube quantum yield. The oxidative functional groups themselves also behave as non-radiative recombination sites that further quench the SWCNT fluorescence.^29^ The resulting decrease in SWCNT fluorescence has long served as a deterrent for using this strategy for optical applications. These limitations, however, can be overcome by engineering chemical handles that inhibit non-radiative recombination, or even enhance radiative recombination, to preserve or increase SWCNT fluorescence, respectively.

### 2.2 Modulating SWCNT optical properties through sp^3^ defects

**Fig. 2:**
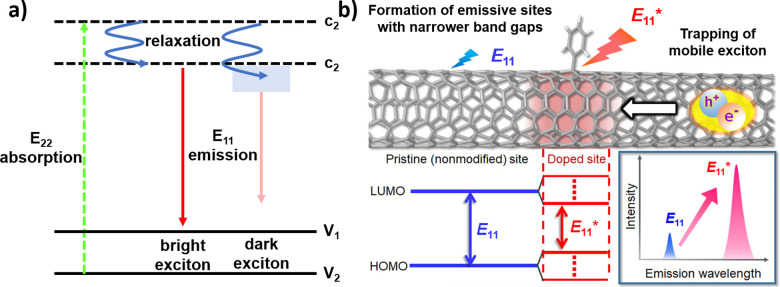
(a) Schematic density of electronic states for a SWCNT. The green arrow depicts the optical excitation to the conduction band. After relaxation (blue arrows), E_11_ excitons are formed that further relax to the valence band (red arrows). In addition to bright excitons (left), the excitation may result in dark excitons (right). (b) Schematic drawing of a modified SWCNT and photoluminescence properties at the pristine and doped sites. Reprinted from ref. 38, with permission from ACS.

The mechanisms for fluorescence preservation and enhancement rely on a foundational understanding of SWCNT exciton dynamics. As shown in Fig. 2a, an E_11_ exciton is formed on E_22_ excitation followed by relaxation.^30^ In addition to bright excitons, the excitation may result in dark excitons that have been shown to ultimately limit the quantum yield of the nanotube.^31^ Unlike conventional fluorophores, SWCNTs have excitons with high binding energies that show exceptional stabilities. Consequently, SWCNT optical transitions are characterized by the behavior of their excitons, rather than free electrons. Furthermore, the resulting excitons can diffuse appreciable distances along the nanotube length prior to recombination. The presence of defects, which include both the nanotube ends as well as oxidative groups along the nanotube, can therefore serve as exciton quenching sites. The non-radiative recombination of bright excitons that occurs at these sites can therefore limit nanotube fluorescence, as discussed in Section 2.1.^32,33^

The fluorescence that is ultimately emitted has been largely attributed to the radiative recombination of the bright E_11_ excitons. This fluorescence varies with the SWCNT chirality and environment, which includes dielectric and electron-withdrawing solvent effects.^32^ With bandgaps on the order of ∼1 eV, the resulting fluorescence emissions often occur in the near-infrared (NIR).^34^ At these wavelengths, the fluroescence emissions are minimally absorbed by biological tissue and visibly opaque fluids. This minimal absorption, along with the relatively deep penetration of low-energy light, motivates the use of SWCNTs for biological and biomedical imaging.^35,36^ In further considering their long-term optical stabilities and sensitivities to their environment, these SWCNTs provide an exceptional framework for continuous optical biosensing.^37^

**Fig. 3:**
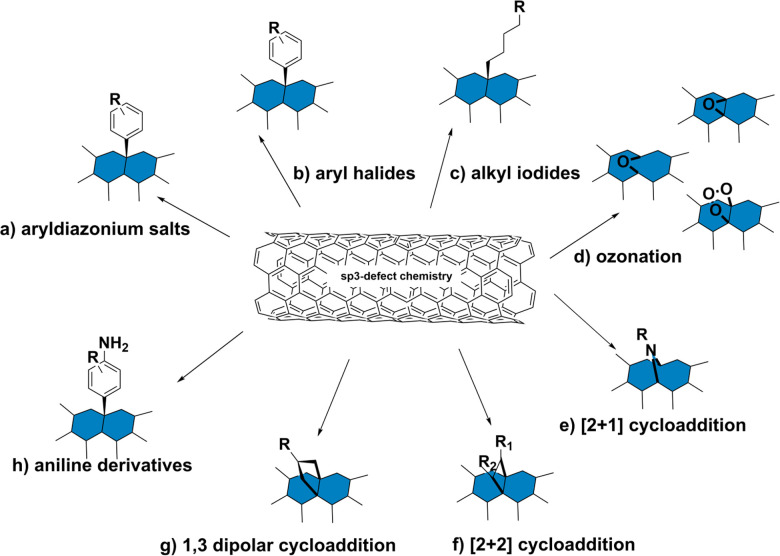
Schematic representation of sp^3^ defect chemistry on SWCNTs including (a) reaction with aryldiazonium salts; (b) reaction with aryl halides; (c) reaction with alkyl iodides; (d) ozonation; (e) [2+1] cycloaddition; (f) [2+2] cycloaddition; (g) 1,3 dipolar cycloaddition; (h) reaction with aniline derivatives.

The preservation of these fluorescence properties is therefore necessary for optical applications. Compared to the acidification described in Section 2.1, where the carboxylic groups may behave as non-radiative recombination sites for diffusing excitons, alternative covalent strategies may result in sp^3^-defect sites that are more favorable to radiative recombination. For example, the SWCNT sidewalls can be covalently modified to produce localized lower energy E_11_^−^ states (Fig. 2b). Radiative recombination at these sites contribute to a red-shifted fluorescence peak (Fig. 2b, lower right). These defect sites could also inhibit the non-radiative decay of excitons to help preserve SWCNT fluorescence.^38^ As a result, the introduction of sp^3^-defects could increase the quantum yield to brighten SWCNTs.^39^ In addition to the advantages in fluorescence, the sp^3^ defect reactions are typically done under milder conditions compared to acid oxidation, which also helps preserve the SWCNT length. Furthermore, as shown in Fig. 3, a collection of chemical handles can be introduced onto the nanotube surface. This diversity brings versatility to the type of cross-linking chemistries that can be subsequently used to immobilize proteins and peptides. These different sp^3^-defect handles are described in greater detail below.^40^

### 2.3 Sp^3^-defect reactions on SWCNT sidewalls

#### 2.3.1 Aryldiazonium salts

One of the most common sp^3^-defect reactions involve the use of aryldiazonium salts (Fig. 3a). As shown in Fig. 3a, these reactions result in the formation of an aryl group covalently bound to the SWCNT surface through a sp^3^–sp^2^ carbon bond between the SWCNT and aryl functional group. As described in previous studies,^39,41^ C_6_H_4_XN_2_^+^BF_4_^−^ (X = –H, –Br, –CH_3_, –COO^−^, –OCH_3_, –N(C_2_H_5_)_2_, –NO_2_ and –C(CH_3_)_3_) are added to a suspension of (6,5) SWCNTs. As the reaction progresses, the functionalization can be monitored through the concomitant decrease in the original (6,5) peak and increase in the red-shifted peak.^39^ These studies reported that reactions with aryldiazonium salt could not only preserve the NIR fluorescence of SWCNTs but also even increase the brightness. The transformation of the carbon from sp^2^ to sp^3^ on the SWCNT sidewalls could also be monitored by Raman spectroscopy.^42^ Since the reaction occurs under mild conditions in the presence of water or buffer, it can be adapted to readily bioconjugate proteins without denaturation.

**Fig. 4:**
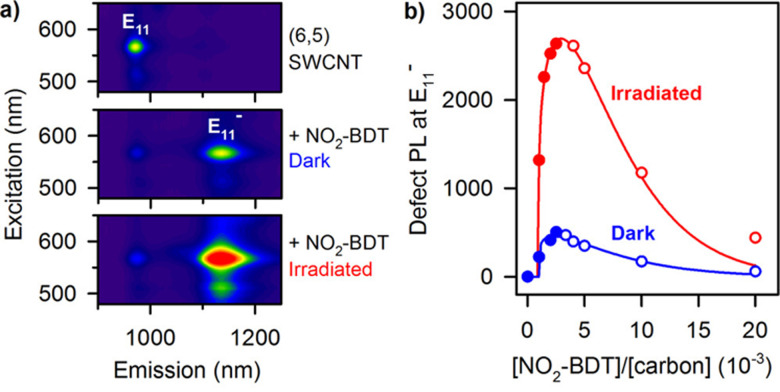
(a) Photoluminescence (PL) excitation–emission maps of (6,5) SWCNTs that are pristine (top), functionalized with p-nitrobenzenediazonium tetrafluoroborate (NO_2_-BDT) under protection from ambient light (middle), and functionalized under irradiation (bottom). (b) Irradiating SWCNTs with light significantly accelerates their functionalization with diazonium salts. Reprinted from ref. 44, with permission from ACS.

Variations of these aryldiazonium reactions can be used to improve yields while also bringing greater chemical diversity to the nanotube surface. For example, previous studies have used diazonium salts with pyrene moieties to form SWCNTs with a dimer of pyrenes on the sidewall.^43^ Additional studies have also focused on optimizing the reaction conditions. For example, Lyndsey R. Powell *et al.* were able to increase loading of the sp^3^-defects through optical excitation (Fig. 4a and b).^44^ The optical excitation was found to enhance the reaction rate, allowing the diazo reagents to react with the SWCNTs over a shorter time with higher yields. Besides directly introducing functional groups for biomacromolecule ligation, the reaction could also introduce sites for conjugate synthesis. In another example, Florian A. Mann *et al.* used fluorenyl methoxycarbonyl (Fmoc)-protected amino acids that were modified with diazonium salt to form a functional handle for peptide synthesis on the SWCNT surface.^45^

#### 2.3.2 Aryl halides and alkyl iodides

**Fig. 5:**
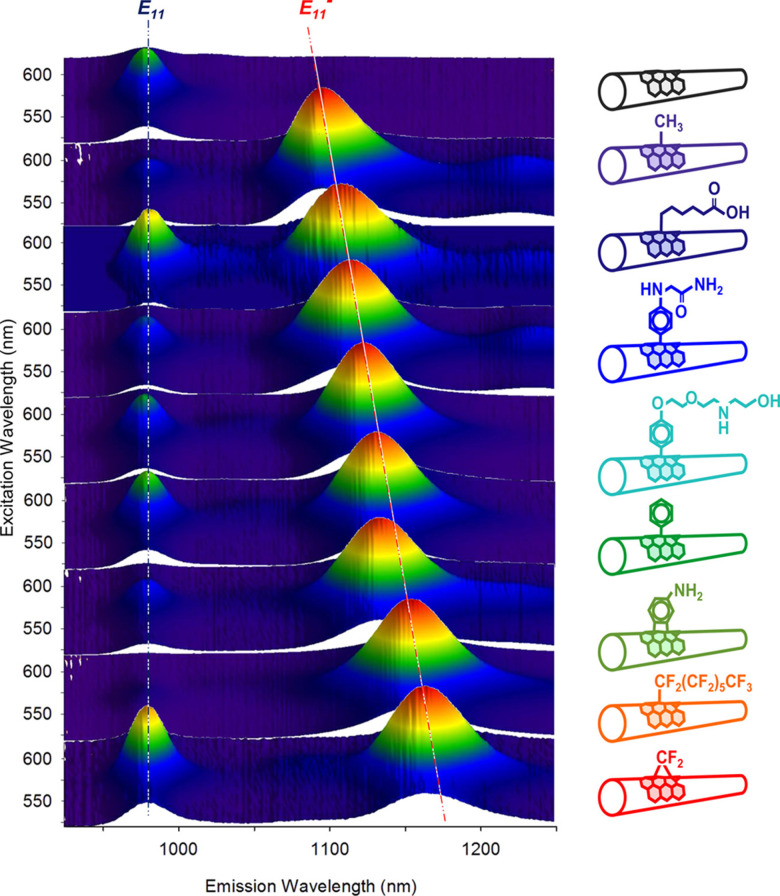
Excitation–emission maps of (6,5) SWCNTs with chemically tailored fluorescent quantum defects. Reprinted from https://pubs.acs.org/doi/full/10.1021/jacs.6b03618, ref. 46 with permission from ACS, further permission related to the material excerpted should be directed to ACS.

Aryl halides can be used to insert functionalized aryl groups onto the SWCNT sidewalls (Fig. 3b). While iodide reagents are primarily used for these reactions, few reports have also used aryl bromide, aryl chloride, or aryl fluoride, though the latter two are less common.^46^ These reactions are found to be driven by light rather than heat. As with the other sp^3^-defect reactions, the fluorescence of the E_11_^−^ peak can be used to monitor the sp^3^-defect formation.^47^ Arylene diiodides^46^ and alkyl iodides (Fig. 3c) have also been used to diversify the chemical functionalization. As shown in Fig. 5, such variations, which include differences in the functional groups on the aromatic rings as well as the length of the alkyl chain and valency of the sp^3^-defect, can influence the shifting and intensity of the E_11_^−^ peak. The underlying mechanism of these reactions was studied by Xiaojian Wu *et al.*, who found these reactions to behave similarly to photocatalyst radical mechanisms.^47^ These reactions showed greater reactivity with reagents that have iodine, which form more stable intermediates. Despite the range of possible functionalities, the aryl halides remain the most widely used reagents for this reaction, as they benefit from commercial availability in addition to mild reaction conditions for protein bioconjugation.

#### 2.3.3 Ozonation with ether and epoxy groups

**Fig. 6:**
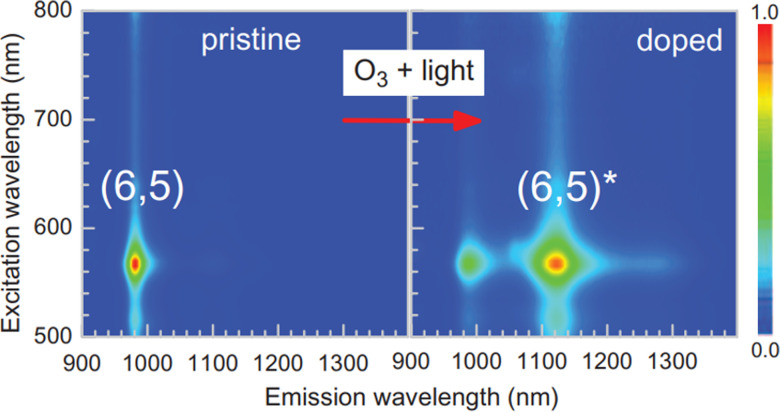
Photoluminescence excitation–emission contour plots from a (6,5)-enriched SWCNT sample before and after treatment. Reprinted from ref. 49, with permission from AAAS.

Although acidification, ozonolysis, and hydrogen peroxidation introduce oxidative sites that can quench mobile excitons (Section 2.1),^48^ alternative oxidation chemistries can create trap sites that enable or even promote radiative recombination. Such trap sites consist of oxygen bridges such as ozonide, epoxide, or ether functionalization (Fig. 3d). Saunab Ghosh *et al.* observed the formation of these oxygen bridges following exposure to ozone and light.^49^ According to Yuhei Miyauchi *et al.*, these trap sites can enhance SWCNT fluorescence by disfavoring nonradiative pathways and by enhancing radiative recombination. The favorable formation of these oxygen bridges is attributed to the controlled exposure to low doses of ozone. Similar to other sp^3^ modifications, the reaction progression can be monitored through the formation of the red-shifted E_11_^−^ peak (Fig. 6).^49,50^

#### 2.3.4 Cycloaddition

Like most organic compounds, the unsaturated carbon bonds of SWCNTs can undergo reactive cycloaddition. Various cycloadditions have been demonstrated on SWCNTs, including the [4+2] cycloaddition (Diels–Alder reaction),^51^ [2+1] cycloaddition (Fig. 3e),^52^ [2+2] cycloaddition (Fig. 3f),^53,54^ and 1,3 dipolar cycloaddition (Fig. 3g).^55,56^ Several of these reactions have been achieved under controlled conditions that preserve the NIR fluorescence.^52–54^ The most prominent of these cycloadditions, the Diels–Alder reaction, occurs between a conjugated diene and a dienophile. In this reaction, the conjugated SWCNTs surface acts as a dienophile. Cécilia Ménard-Moyon *et al.* were among the first to demonstrate a Diels–Alder reaction between different dienes and unmodified SWCNTs.^57^ In this study, the SWCNTs showed differences in reactivity with linear diene (2,3-dimethoxy-1,3-butadiene) and cyclodiene (9,10-dimethylanthracene) using a cobalt catalyst at high temperatures. These reactions served as a basis for modified Diels–Alder reactions with SWCNTs that use more complex structures (polymers with diene groups), different catalysts, and different solvents.^58^ The Diels–Alder chemistry is currently used for the synthesis of multifunctional SWCNT-polymer composites.

In addition to the Diels–Alder reaction, SWCNT cycloaddition can also be achieved through 1,3 dipolar addition. For example, Vasilios Georgakilas *et al.* demonstrated the 1,3 dipolar addition of SWCNTs using azomethine ylides prepared with aldehyde and glycine derivatives.^56^ The cycloaddition was confirmed by nuclear magnetic resonance (NMR) spectroscopy, Raman spectroscopy, and transmission electron microscopy (TEM). Although the authors observed a decrease in the NIR emissions following the chemical modification, this addition can be used to introduce long-chain polymers and conjugates commonly required for biomedical applications.

**Fig. 7:**
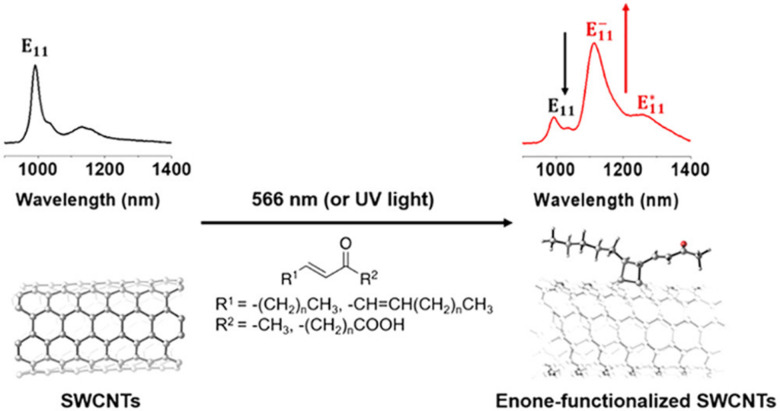
Fluorescence emission spectra of SWCNTs before (left) and after (right) [2π+2π] photocycloaddition of enones. Reprinted from ref. 54, with permission from ACS.

Of particular interest for fluorescent SWCNT conjugation are the [2+1] and [2+2] additions, which were among the first cycloadditions to yield an increase in SWCNT fluorescence. Antonio Setaro *et al.* demonstrated a [2+1] cycloaddition reaction between electron-poor aromatic nitrenes originating from azido compounds and SWCNTs. Compared to the cycloaddition reactions described above that required high temperatures and a metal catalyst, these [2+1] cycloadditions could react at room temperature in the absence of an added catalyst. In these reactions, the first step is succeeded by a ring-opening and rehybridization step that rebuilds the conjugation system. These reactions could further be expanded to other substrates, such as spiropyran or thiol-substituted nitrenes.^52^ Similarly, [2+2] cycloaddition can also be achieved using oxidation products of linoleic acid.^54^ The enone in linoleic acid could react with a conjugated bond on the SWCNT surface in the presence of a photocatalyst under UV or 566 nm illumination (Fig. 7). Reactivity was also observed with substrates containing a carbonyl group in the absence of a carbon double bond, though to a lesser extent than enone-containing substrates. Although only a limited number of cycloaddition studies to date have examined the effects on SWCNT fluorescence, the variety of substrates enabled by this chemistry has been crucial for diversifying sp^3^-defects.

#### 2.3.5 Reaction with aniline derivatives

Among the different sp^3^ moieties that could be introduced to the surface of SWCNTs, amino groups benefit from reactivity that can be used for further bioconjugation chemistry. Amino groups can serve as ligation handles for polymers and biomacromolecules. Their reactivity with aromatic rings is also useful for creating a distinct physiochemical signature for characterization. Among this class of reagents, anilines in particular have shown strong promise for SWCNT biochemical characterization and application (Fig. 3h). Sp^3^ reactions between aniline and SWCNTs result in the formation of two new E_11_^−^ peaks.

**Fig. 8:**
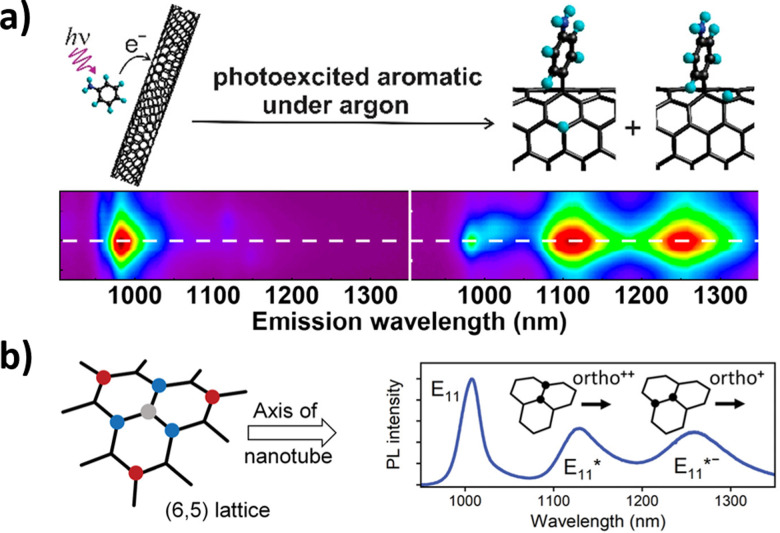
(a) Excitation–emission plots of CoMoCAT SWCNTs in 1% aqueous sodium dodecyl sulfate (SDS) in the presence of organic aromatics under argon. Reprinted from ref. 60, with permission from the ACS. (b) Binding configurations of substituents to a (6,5) nanotube lattice fragment. The formation of the initial sp^3^ carbon atom (grey) is followed by a binding event at the *ortho* (blue) or *para* (red) positions, all of which create different defect states. Binding to the different *ortho* positions results in the formation of different E_11_* and E_11_*^−^ photoluminescence (PL) emission peaks for the (6,5) SWCNT. Reprinted from ref. 40, with permission from Wiley-VCH GmbH.

These peaks are believed to correspond to mechanisms resulting in products with different configurations of hydrogen atoms.^59,60^ Yu Zheng *et al.* identified oxygen as a critical component in these reactions. The reaction is believed to proceed through radical formation, with the hydrogen reacting with both the *ortho* and *para* positions of the aryl binding sites.^60^ These distinct binding sites are believed to form two products that are captured by different NIR fluorescence emissions. Aniline defect sites have also been achieved by Simon Settele *et al.*, who performed the reaction under light excitation. The resulting products show hydrogen reacting at the *ortho* positions, but with different configurations relative to the SWCNT axis (Fig. 8). The authors hypothesized that one of the products was formed from a light-induced radical mechanism while the other from nucleophilic attack. The distinct NIR emissions that emerge from the different aniline products is promising for engineering multi-chromic optical sensors.

## 3. Bioconjugation reactions with proteins

### 3.1 Reactive functional handles on proteins

**Fig. 9:**
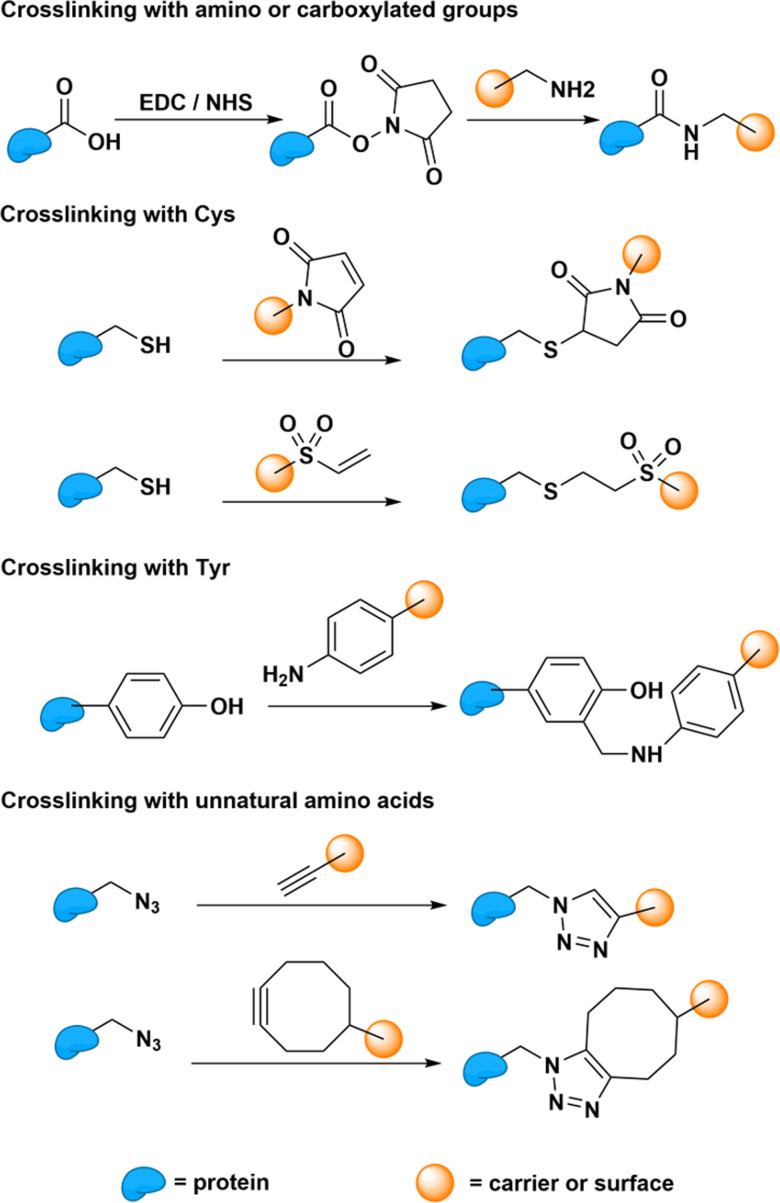
Bioconjugation reaction between different protein residues (cysteine, Cys; tyrosine, Tyr) and carriers (*e.g.*, SWCNTs and polymers) or surfaces (*e.g.*, silica).

Protein bioconjugation reactions can occur either at the terminal or at targeted residues within the sequence (Fig. 9). Terminal protein reactions commonly achieve crosslinking through 1-ethyl-3-(3-dimethylaminopropyl)carbodiimide (EDC)-mediated coupling. These reactions are often carried out in the presence of N-hydroxysuccinimide (NHS) to increase the stability of the intermediate and enhance reactivity. Sulfo-NHS can also be used under conditions that require increased NHS solubility.^61^ The reaction results in the formation of amide bonds between amino and carboxylated groups. This reaction could therefore be used to conjugate proteins to desired carriers and surfaces, such as SWCNTs, polymers, or silica, from either their N- or C-terminal.^62–64^ The ubiquitous presence of amino and carboxylate groups therefore allows this reaction to be readily applied to most proteins. However, the presence of competing amino groups on non-terminal lysine residues and carboxylated groups on non-terminal aspartic acid and glutamic acid residues limit the selectivity of the reactions at the N-terminal and C-terminal sites, respectively.

Reactions that require site-specific conjugation must therefore rely on the bioconjugation of less common functional handles. These site-specific reactions enable greater control over the protein orientation on the carrier or surface. This greater control and homogeneity in protein orientation may improve enzyme–carrier interactions through increased electron, energy transfer, or other transduction mechanisms that may increase the response of the sensor.^65^ Cysteine residues, which are uncommon on natural proteins, are therefore often used for site-specific conjugation. These residues harbor thiol groups that are not found on any other natural residues. Through Michael addition, these cysteine thiol groups can conjugate with carriers and surfaces that carry a double bond.^66^ For proteins that lack any natural cysteine residues, tyrosine could also be targeted for bioconjugation through Mannich-type reactions.^67^ However, compared to cysteine, these tyrosine conjugations show less reactivity. Therefore, researchers often opt to introduce artificial cysteine residues for bioconjugation through bioengineering. Beyond artificially introducing natural residues, this strategy could also be used to introduce unnatural amino acids capable of specific and reactive “click” reactions.^68^ Whether natural or artificial, these protein functional groups can therefore subsequently serve as anchoring sites for covalent bioconjugation onto SWCNTs.

### 3.2 Bioconjugation strategies between proteins and SWCNTs

The aforementioned reactions yield chemical handles that can be used to immobilize proteins. Protein immobilization is necessary for many biomedical and biological applications, including drug delivery, electrical signalling, and optical sensing.^3,12^ One of the most common approaches for immobilization is protein adsorption *via* non-covalent interactions. This immobilization strategy relies on surface residues capable of π–π stacking, hydrophobic, and van der Waals interactions with SWCNTs. Although relatively facile, this non-covalent strategy results in heterogenous conjugates with various configurations and protein loading. Moreover, these conjugates remain relatively susceptible to long-term instability, particularly under denaturing *in vivo* conditions. These disadvantages motivate the development and application of covalent-based immobilization strategies.

One approach to covalently immobilizing proteins is to introduce functional groups on SWCNTs that can be used to conjugate proteins or protein derivatives. Current studies have largely focused on covalent conjugation between carboxylated SWCNTs and the amino groups on proteins, as described above. Carboxylated SWCNTs are activated by EDC or EDC/NHS to form a semi-stable intermediate that can react with the free amino groups on a protein surface.^69,70^ This immobilization strategy has been used to immobilize hydroxyquinol dioxygenase with a relatively high yield (52.1%) and enzyme activity of up to 64.7%.^69^ Despite the relative ease of the SWCNT carboxylation and the versatility of this approach to a variety of wildtype enzymes, this strategy often results in the complete or partial denaturation of enzyme structure and activity. For this reason, long polymer chains have been used to minimize inactivation by increasing the distance between the proteins and the SWCNTs.^71^ Another strategy to increase the activity is to link the SWCNTs with an active group, such as maleimide derivatives,^72^ that the proteins could more readily access. Though polyethylene glycol (PEG)-based polymers are the most widely applied in these ligations,^73^ additional polymers have since been adapted to these strategies. In addition to carboxylated SWCNTs, other functional handles can also be used to target specific protein groups. For example, the 1,3 dipolar cycloadditions of ylides can introduce amino groups that react with the carboxylic groups on proteins through a simple condensation reaction. Another example is the reaction of amino-modified SWCNTs with a terminal end of a *N*-succinimidyl-3-maleimidopropionate linker, which was used to conjugate a cysteine protein residue at the other terminal end.^72^ An alternative strategy developed by Mark Freeley *et al.* relied on DNA hybridization between a single-stranded DNA–protein conjugate and a complementary DNA strand that was covalently linked to the SWCNT.^74^ In this case, the covalent DNA was conjugated to the end of the SWCNT. Though the NIR fluorescence was not characterized in this study, the placement of the covalent link is expected to have minimal effects on the SWCNT fluorescence, since the SWCNT ends themselves already act as exciton quenching sites.

**Fig. 10:**
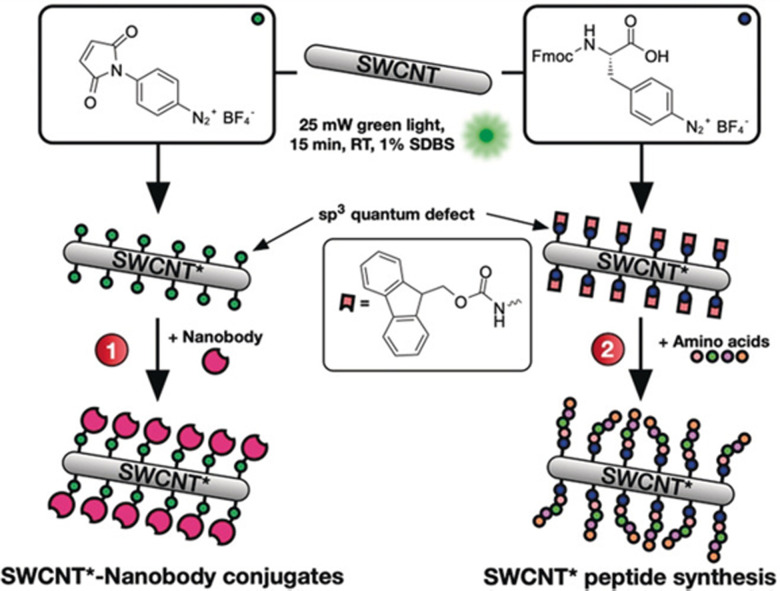
Defects as a generic handle to functionalize SWCNTs in sodium dodecylbenzenesulfonate (SDBS) at room temperature (RT). Various aryl defects are introduced onto the nanotube's sidewall and used to conjugate biomolecules such as (1) nanobodies or (2) amino acids for the direct growth of peptide chains on the modified SWCNT*. Reprinted from ref. 45, with permission from Wiley-VCH GmbH.

In addition to the linker strategies described above, modified proteins or amino acids may also directly react with SWCNT surfaces. Suzanne K. Thomas *et al.* used photocatalysis to conjugate SWCNTs to green fluorescent protein (GFP) through a genetically encoded phenyl azide group. Although the effects on the SWCNT NIR fluorescence remain largely unexplored, several studies have made efforts to characterize the optoelectronic properties of the protein and SWCNT after conjugation.^75^ For example, a later study found that the SWCNT maintains its semiconducting properties just as the GFP is able to sustain its fluorescence after conjugation. These findings enabled the construction of a bio-optoelectronic transistor based on this covalent strategy.^76^ In another example, Florian A. Mann *et al.* employed the aryldiazonium salt-based sp^3^-defect reaction to introduce maleimido groups or Fmoc-protected amino acids (Fig. 10).^45^ The resulting maleimido groups on the SWCNTs could conjugate to the thiol groups on nanobodies to form nanobody conjugates. Meanwhile, the SWCNTs modified with the Fmoc-protected amino acids was used to initiate peptide synthesis on the SWCNT surface. Interestingly, both strategies resulted in the formation of directly conjugated SWCNTs that show sustained fluorescence emissions.

## 4. Purification and characterization of protein–SWCNT conjugates

The formation of these SWCNT conjugates is often followed by sample purification to remove unbound or non-specific protein conjugates. Even in the case of covalent strategies, these reactions can yield mixtures of covalently linked proteins as well as non-specifically bound non-covalent conjugates. The presence of catalysts, coupling reagents, and activators can favor the kinetic formation of covalent conjugates over non-covalent conjugates, particularly over shorter reaction times.^14^ As the covalently conjugated SWCNTs tend to show greater stability, these conjugates can demonstrate higher tolerance to harsher or more extensive washing. Thus, the primary approach to separating reacted and unreacted conjugates is through repeated washing and dialysis. Though this approach can be used to improve the isolation of covalent conjugates, they often yield residual amounts of non-covalent conjugates that require additional purification steps.^75^

**Fig. 11:**
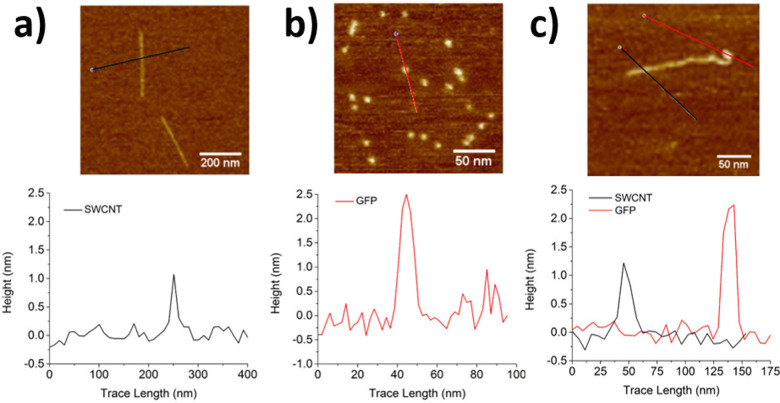
AFM images and corresponding height profiles for (a) ssDNA/SWCNTs, (b) individual GFP^SA^ (SA = short axis), and (c) SWCNT-GFP^SA^ hybrids. The same heights were observed for GFP^LA^ (LA = long axis) and its hybrids. *Z*-scales = 6 nm. Reprinted from ref. 81, with permission from ACS.

Several methods can be used to characterize the resulting SWCNT conjugates. The Bradford and bicinchoninic acid (BCA) assays are used to evaluate protein loading on SWCNTs.^77,78^ Protein loading can also be determined by calculating the decrease of protein concentration in solution during the reaction as measured with UV-vis spectroscopy and high-performance liquid chromatography (HPLC). However, protein degradation may also contribute to further decreasing the measured concentration. The modification of the SWCNT surface can be characterized using Fourier-transform infrared (FT-IR), Raman, and NMR spectroscopies.^76,79^ Whereas FT-IR spectroscopy can be used to characterize most products, 2D-NMR spectroscopy can offer a more accurate means of characterizing the conjugation between small proteins or peptides with SWCNTs. X-Ray photoelectron spectroscopy (XPS) could also be used to confirm the immobilization of proteins on SWCNTs, providing both qualitative and quantitative information on the immobilization.^80^ The conjugated structure can also be characterized through microscopic imaging. For example, TEM and scanning electron microscopy (SEM) can differentiate unmodified SWCNTs that appear as homogeneous long fibers from SWCNT conjugates that appear as nonuniform structures with aggregates.^69^ Atomic force microscopy (AFM) can also provide quantitative information on the conjugate structure under less denaturing conditions.^75^ SWCNTs that are unmodified or only activated by small molecules have a height about 1 nm. By contrast, protein–SWCNT conjugates show larger heights of over 2 nm (Fig. 11).^81^ In addition to structural changes, the protein immobilization can also change the charge distribution of the SWCNT surface. This change can be characterized through zeta potential measurements. Together with the spectroscopic confirmation of the bond formation and microscopic analysis of the structures, these techniques can be used in combination to validate protein immobilization.

## 5. Applications of protein–SWCNT conjugates

### 5.1 Safety of SWCNTs for biomedical applications

The novel electrical and optical properties of SWCNTs justify the remarkable potential of SWCNTs for biomedical applications. These applications, however, warrant additional considerations on the safety and biotoxicity of SWCNT conjugates.^82^ Concerns on the toxicity of SWCNTs largely stem from the production of reactive oxygen species that may lead to cell apoptosis and inflammation.^83^ The greatest risk, however, concerns SWCNTs in the dry state. The light, SWCNT fibers closely resemble those of asbestos that have been shown to lead to lung cancer and mesothelioma when inhaled. However, the solubilized SWCNTs that are often used for biomedical applications are not readily airborne, mitigating the risk of inhalation. The risk of toxicity is further decreased with dispersity, as well-dispersed SWCNTs show greater biocompatibility compared to SWCNT aggregates.^84^ The functionalization of the SWCNT surface also plays a critical role in modulating toxicity. Biocompatible polymer coatings like PEG, for example, can be used to passivate unwanted SWCNT surface interactions.^85^ Modifications on SWCNTs could also influence the toxicity of the SWCNTs themselves. Notably, the same carboxylation strategy used to synthesize cytotoxic carboxylated SWCNTs can also be used to diminish toxicity by increasing the loading of carboxylated groups. These groups can decrease toxicity by increasing the hydrophilicity and dispersion of the conjugates.^86^ In this sense, protein functionalizations themselves can be used to mitigate toxicity. Such functionalizations can be achieved using abundant and typical blood proteins, such as bovine fibrinogen, immunoglobulin, transferrin, and bovine serum albumin (BSA).^87^ In summary, the biocompatibility of these structures lies squarely with the functionalization strategies for encapsulating SWCNTs.^88^ These strategies include those that may very well build on the protein chemistries discussed herein.

### 5.2 Enzyme applications

**Fig. 12:**
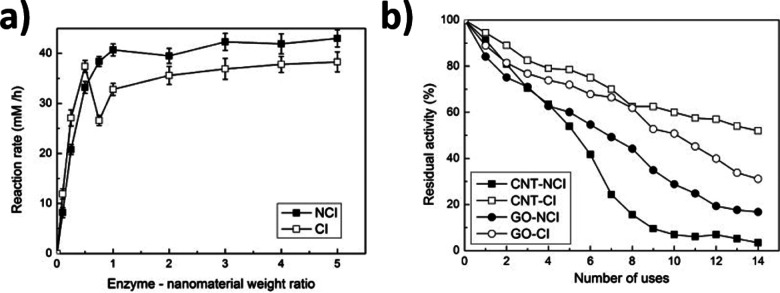
Effect of the enzyme to nanomaterial weight ratio on the butyl caprylate synthesis catalyzed by *Candida Antarctica*. (a) lipase B (CalB) noncovalent immobilization (NCI) and covalent immobilization (CI) on amine-functionalized carbon nanotube (CNT). (b) Reusability of CalB in *n*-hexane at 60 °C when the enzyme is covalently or non-covalently immobilized on amine-functionalized carbon nanotubes or graphene oxide (GO) (standard deviation was less than 5% in all cases). Reprinted from ref. 80, with permission from Elsevier.

Enzyme immobilization has advantages in stability, recyclability, and product purification.^4,89^ Compared to solid surfaces, immobilization on nanostructures may also increase the loading yield and reactivity of the enzymes.^90^ SWCNTs have been used as enzyme carriers that benefit from a simple structure and chemical bonds that are amenable to further engineering. Ioannis V. Pavlidis *et al.* found that the covalent conjugation of hydrolases could increase the reactivity and stability of free enzymes.^80^ Compared to enzymes loaded on supports like graphene oxide or non-covalently immobilized on SWCNTs, the covalent conjugates showed enhanced physiochemical properties (Fig. 12a). The covalent conjugates also exhibit higher operational stability, retaining over 60% of enzyme activity after 14 heating–cooling cycles (Fig. 12b). This stability motivates the application of nanomaterial bioconjugates for industrial catalysis.

Beyond advantages in stability and loading, immobilization strategies that retain the SWCNT NIR fluorescence could further benefit applications in analytical optical detection. Previous studies have shown that the immobilization of proteins, both covalent and non-covalent, can trigger a change in SWCNT fluorescence.^45,91^ The extent of the immobilization can therefore be monitored in real-time without the use of added fluorescent or absorption dyes simply by tracking the NIR fluorescence. Compared to techniques such as XPS, NMR or FT-IR, this approach obviates the need for additional sample preparation. Importantly, the NIR fluorescence can also be used to monitor the activity of the enzyme on the SWCNT. For example, SWCNT fluorescence can be used to monitor the activity of polymerases and restriction enzymes on substrates such as single-stranded and double-stranded DNA.^92,93^ This approach can also be used to detect the consumption of a substrate catalyzed by the enzyme. For example, Vitalijs Zubkovs *et al.* developed an optical sensor for glucose based on the enzymatic activity of glucose oxidase (GOx) (see Section 5.4).^94^

### 5.3 Pharmaceutical applications

Protein–SWCNT conjugates are also important for pharmaceutical applications, including immunological studies. Fields like immunology hinge on the ability to explore interactions between receptors and bioanalytes. The *in vitro* study of these interactions often requires an immobilization scaffold for presenting the antigen or antibody. This need is exacerbated for immunology frameworks that require multivalent structures. Functionalized SWCNTs have been used for such molecular immobilizations and delivery applications.^95^ SWCNTs have been used as carriers for small molecules including antibiotics, fluorescent probes, and even antitumor drugs. Similar design strategies apply to protein–SWCNT conjugates developed for immunological assays. For example, Davide Pantarotto *et al.* synthesized conjugates of SWCNTs with VP1 protein fragments.^73^ The protein fragments were linked to the surface of the SWCNTs with double-armed linkers to form a multivalent structure. This conjugate exhibited a moderate binding affinity to antibodies *in vitro*. Furthermore, the mice immunized by these conjugates had a high level of antibody expression compared to mice immunized by only the protein fragments. In these applications, covalent SWCNT chemistry can be used to control the relative positions and orientations of immobilized complexes for multivalent vaccine and drug design. The fluorescent SWCNT properties can similarly be used to monitor binding and release interactions for these pharmaceutical applications.

### 5.4 Optical imaging and sensing

**Fig. 13:**
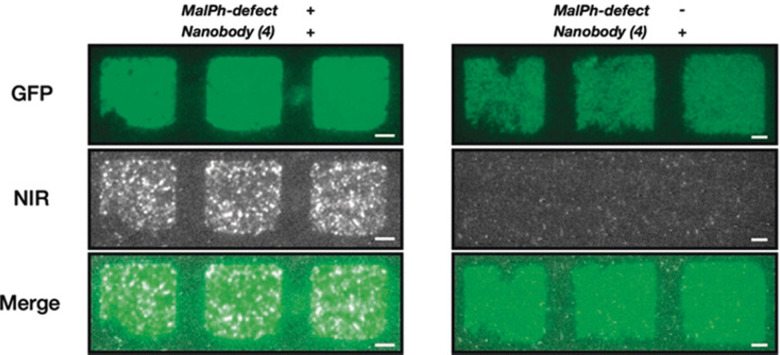
Pattern of GFP on a glass surface incubated with SWCNT*-GBP. The colocalization shows retained functionality of the nanobody even after covalent conjugation to the SWCNT with (N-maleimido)phenyl quantum defects (MalPh-defect). Scale bars = 5 μm. Reprinted from ref. 45, with permission from Wiley-VCH GmbH.

As described above, SWCNTs have NIR fluorescence that is useful for optical bioimaging and biosensing. The preservation of this SWCNT fluorescence has often been the focus of previous optical sensing studies that rely on non-covalent immobilization strategies.^35^ Covalent strategies, on the other hand, have shown more limited application due to the resulting quenching of the NIR signal that is typically observed following acid treatment. Recent advancements in sp^3^ defects that can preserve or enhance this NIR fluorescence allow alternative immobilization strategies for bioimaging and biosensing. Most recently, these defect sites were introduced to DNA-wrapped SWCNTs that showed optical sensitivity toward desired analytes.^96^ These sites have also been used for the optical detection of protein interactions in addition to the enzymatic interactions described in Section 5.2.^97^ Triazine-modified SWCNTs have been conjugated to PEG-biotin, which has a strong binding affinity to avidin proteins. The optical response of these constructs was monitored with the addition of BSA-biotin. In another application, sp^3^-defect SWCNTs were modified with peptides or GFP-binding nanobodies (GBP). The GBP-SWCNT conjugates could bind GFP, resulting in the visible and NIR immobilized pattern observed in Fig. 13.^45^

### 5.5 Monitoring charge transfer on electrodes

**Fig. 14:**
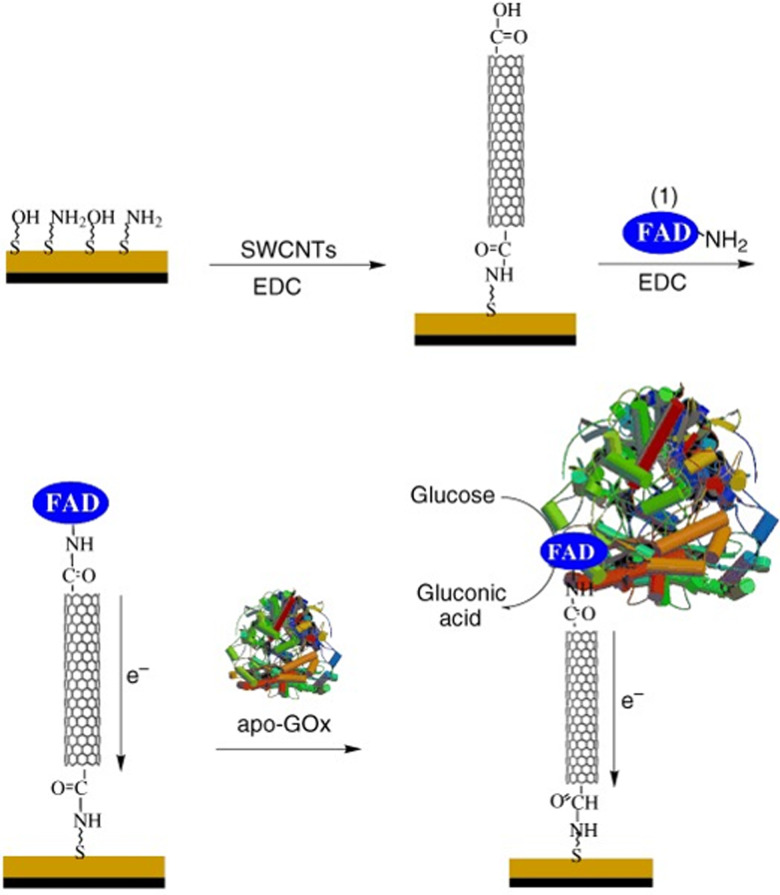
Assembly of electrode consisting of GOx immobilized onto a SWCNT *via* flavin adenine dinucleotide (FAD). Reprinted from ref. 100, with permission from Wiley-VCH GmbH.

In addition to optics, electronic applications rely heavily on effective immobilization strategies. In these applications, SWCNTs provide conductivity, mechanical stability, and a large surface for analyte detection. Enzymatic activity, for example, can be monitored electronically *via* energy and charge transfer.^98,99^ Since covalent linkage between enzymes and conducting electrodes can increase the efficiency of charge transfer (Fig. 14),^100^ the conjugation may contribute to a more pronounced electronic signal.^101,102^ Conventional approaches for enzyme loading include adsorption, supramolecular recognition, and π–π stacking interactions. Compared to optical applications, however, electronic applications have historically benefitted from a broader development of various covalent conjugation chemistries.^103^ For these applications, the decrease in NIR fluorescence plays no substantial role in the device operation, which only stands to benefit from the greater stability and enhanced energy transfer from covalent conjugation. The EDC conjugation method, for example, has been used to immobilize lactate oxidase onto SWCNT electrodes.^18^ This study showed that while covalent and non-covalent conjugates showed comparable sensitivity, linear range, and detection limits, the covalent conjugates exhibited higher stability. Furthermore, the higher stability conferred by the covalent conjugates enable more complex operations, such as multi-step alignments commonly used in the construction of sensor arrays.^104,105^ These demonstrations thus motivate the application of recent sp^3^-defect chemistries to electronic devices. The NIR monitoring of charge transfer provides an orthogonal, yet complementary, approach to studying electron transfer mechanisms. Such applications therefore stand to benefit from the distinct optoelectronic properties offered by these new chemistries.

### 5.6 Outlook on SWCNT–protein conjugation chemistries

The past years have seen a significant advancement in the precision in introducing these sp^3^ chemistries. Opportunities for further advancement lie with broadening the diversity of these chemistries. The chemistries described above are largely limited to the functional groups summarized in Fig. 3 and 9. Several of the reactive pairs rely on overlapping functional groups. Greater diversity in reactive functional pairs that do not share cross-reactivity is crucial for enabling bioorthogonal reactions. This bioorthogonality is especially helpful for applications that require the immobilization of multiple proteins, multi- or single-step immobilization patterns, or multimodal optical monitoring of several reactions simultaneously. In addition to the reaction pairs, the diversity of immobilized proteins also limits current applications in the field. Despite their promise, sp^3^ defect chemistries have largely been limited to immobilizing fluorescent, non-reactive proteins. The demonstration of a broader range of proteins with diverse functionalities may provide key insights for new optical applications as well as new mechanisms for studying SWCNT photophysics. Such proteins may include, for example, receptors of biomolecules in addition to enzymes with novel activities and therapeutic proteins. The broader applicability of these chemistries to new proteins hold promise for new biomedical applications. Beyond experimental advancements, additional opportunities lie on the theoretical front. The tradeoff between optical efficiency and SWCNT loading of the functional groups is often unpredictable and characterized, at best, empirically. The ability to predict and quantify the effects of functional handles on NIR fluorescence will be key to unlocking new chemistries and reaction conditions with optimized performances.

## 6. Conclusion

The recent advancements in sp^3^-defect chemistry show strong promise in overcoming conventional challenges in biomolecule immobilization on SWCNT surfaces. In particular, they open the doors to multiplexed covalent strategies that are in the infancy of their exploration. Of particular interest is the distinct optoelectronic properties enabled by these new chemistries, which can be exploited for optical sensing and imaging applications. In these design strategies, the SWCNT surface can be modified with functional handles that serve as covalent anchors for linkers or protein residues. These strategies are therefore able to combine the advantages of covalent stability along with the retention of NIR fluorescence. Importantly, the colocalization of the immobilized biomolecule on a localized exciton site allows not only a fundamental exploration of SWCNT photophysics but also improvements in optoelectronic applications.

## Conflicts of interest

There are no conflicts to declare.

## Supplementary Material
